# Trial protocol OPPTIMUM– Does progesterone prophylaxis for the prevention of preterm labour improve outcome?

**DOI:** 10.1186/1471-2393-12-79

**Published:** 2012-08-06

**Authors:** Jane E Norman, Andrew Shennan, Phillip Bennett, Steven Thornton, Stephen Robson, Neil Marlow, John Norrie, Stavros Petrou, Neil Sebire, Tina Lavender, Sonia Whyte

**Affiliations:** 1 University of Edinburgh MRC Centre for Reproductive Health, The Queen’s Medical Research Institute, 47 Little France Crescent, Edinburgh, EH16 4TY, UK; 2 Women's Health Academic Centre, King’s Health Partners, 10th floor North Wing, St.Thomas' Hospital, London, SE1 7EH, UK; 3 Imperial College Faculty of Medicine, Institute for Reproductive and Developmental Biology, Hammersmith Hospital Campus, Du Cane Road, London, W12 0NN, UK; 4 Peninsula College of Medicine and Dentistry, Peninsula Medical School, Barrack Road, Exeter, EX2 5DW, UK; 5 Institute of Cellular Medicine, Uterine Cell Signalling Group, 3rd Floor, William Leech Building, The Medical School, Newcastle University, Newcastle, NE2 4HH, UK; 6 Institute for Women’s Health, University College London, Room 244, Medical School Building, 74 Huntley Street, London, WC1E 6AU, UK; 7 Centre for Healthcare Randomised Trials (CHaRT), Health Services Research Unit, 3rd Floor Health Sciences Building, University of Aberdeen, Foresterhill, Aberdeen, AB25 2ZD, UK; 8 University of Warwick Division of Health Sciences, Warwick Medical School, The University of Warwick, Coventry, CV4 7AL, UK; 9 Histopathology Department, Department of Paediatric Laboratory Medicine, Level 3, Camelia Botnar Laboratories, Great Ormond Street Hospital, London, WC1N 3JH, UK; 10 School of Nursing, Midwifery and Social Work, The University of Manchester, Manchester, UK; 11 University of Edinburgh MRC Centre for Reproductive Health, c/o Simpson Centre for Reproductive Health, Royal Infirmary, 51 Little France Crescent, Edinburgh, EH16 4SA, UK

## Abstract

**Background:**

Preterm birth is a global problem, with a prevalence of 8 to 12% depending on location. Several large trials and systematic reviews have shown progestogens to be effective in preventing or delaying preterm birth in selected high risk women with a singleton pregnancy (including those with a short cervix or previous preterm birth). Although an improvement in short term neonatal outcomes has been shown in some trials these have not consistently been confirmed in meta-analyses. Additionally data on longer term outcomes is limited to a single trial where no difference in outcomes was demonstrated at four years of age of the child, despite those in the “progesterone” group having a lower incidence of preterm birth.

**Methods/Design:**

The OPPTIMUM study is a double blind randomized placebo controlled trial to determine whether progesterone prophylaxis to prevent preterm birth has long term neonatal or infant benefit. Specifically it will study whether, in women with singleton pregnancy and at high risk of preterm labour, prophylactic vaginal natural progesterone, 200 mg daily from 22 – 34 weeks gestation, compared to placebo, improves obstetric outcome by lengthening pregnancy thus reducing the incidence of preterm delivery (before 34 weeks), improves neonatal outcome by reducing a composite of death and major morbidity, and leads to improved childhood cognitive and neurosensory outcomes at two years of age. Recruitment began in 2009 and is scheduled to close in Spring 2013. As of May 2012, over 800 women had been randomized in 60 sites.

**Discussion:**

OPPTIMUM will provide further evidence on the effectiveness of vaginal progesterone for prevention of preterm birth and improvement of neonatal outcomes in selected groups of women with singleton pregnancy at high risk of preterm birth. Additionally it will determine whether any reduction in the incidence of preterm birth is accompanied by improved childhood outcome.

**Trial registration:**

ISRCTN14568373

## Background, including rationale and any previous systematic review(s)

Preterm birth is a global problem, with a prevalence of 8 to 12% depending on location [1]. Around 75% of preterm birth follows spontaneous preterm labour, sometimes preceded by preterm premature membrane rupture [2]. Babies born preterm are at increased risk of a variety of adverse short term (neonatal) and long term complications, including neurodevelopmental disability. Women with a previous preterm birth (especially those women who delivered before 34 weeks following spontaneous preterm labour), women with a short cervix in early pregnancy [3], and women with a previous cone biopsy or laser loop excision to the cervix [4] are at all at increased risk of spontaneous preterm birth.

Several large trials and meta-analyses have shown progesterone to be effective in preventing or delaying preterm birth in selected high risk women (short cervix or previous preterm birth) with singleton pregnancy [5-11]. There is data that both intramuscular 17 hydroxyprogesterone caproate and vaginal progesterone are effective in preventing preterm birth. OPPTIMUM likewise is investigating the efficacy of progesterone in women at elevated risk of preterm birth, but will crucially address longer term childhood developmental outcomes.

Although an improvement in short term neonatal outcomes has been shown in some trials [6-8] these have not consistently been confirmed in meta-analyses [9,10]. Data on longer term outcomes in singletons is limited to follow up of babies of women in the Meis trial, where 80% of babies were assessed by questionnaire at a mean age of four years. No differences in childhood outcomes were demonstrated, despite the progesterone group having a lower incidence of preterm birth [12].

The mechanisms of action of progesterone are somewhat uncertain although a direct inhibitory effect on the processes of parturition seem likely [13]. Additionally progesterone could exert anti-inflammatory properties [13] and/or direct CNS protective effects [14], which could help to reduce the risk or severity of long term neonatal problems.

If the prevention of preterm birth is accompanied by a reduction in the complications of prematurity, then *in utero* progesterone should be predicted to have long term beneficial effects. Harmful long term effects are however also possible. Although direct teratogenic effects of progesterone are unlikely, there could be adverse effects of keeping the fetus in utero in a compromised intrauterine environment where infection or inflammation is present. Caution is therefore warranted before progesterone use becomes widespread, particularly since drugs (including antibiotics and estrogens) thought to be beneficial in women at risk of preterm birth have now been demonstrated to have long term adverse effects [15-17].

The OPPTIMUM study is designed to determine whether progesterone prophylaxis to prevent preterm birth has long term neonatal or childhood benefit. Specifically it will study whether, in women at elevated risk of preterm labour, prophylactic vaginal natural progesterone, 200 mg (compared to placebo), daily from 22 – 24 weeks up to 34 weeks gestation, improves obstetric outcome by lengthening pregnancy thus reducing the incidence of preterm delivery (before 34 weeks), improves neonatal outcome by reducing a composite of death and major morbidity and leads to improved childhood cognitive and neurosensory outcomes at two years of age.

The OPPTIMUM study began recruiting in January 2009. Since then, one large, and several other smaller studies have reported the effect of progesterone, either as vaginal progesterone or as intramuscular 17 hydroxyprogesteronecaproate. None fully addresses the crucial question regarding long term outcome (childhood development at 2 years). Additionally, we note that the US Food and Drug Administration (FDA) has indicated that long term childhood outcome data are required to determine the clinical benefits and risks of 17 hydroxyprogesteronecaproate for the prevention of preterm birth in women with a previous preterm birth._._^a^ An FDA panel also recently ruled that data supplied on vaginal progesterone gel “do not [yet] support the efficacy of progesterone gel compared with placebo in reducing the risk of preterm births before 33 completed weeks of gestation among women with a short cervical length”.^b^ We believe that OPPTIMUM will address these important evidence gaps about the efficacy of vaginal progesterone and any childhood effects of progestogens in general.

## Methods

### Aim(s)

The aim of the OPPTIMUM study is to determine whether, in women at high risk of preterm labour, prophylactic vaginal natural progesterone, 200 mg daily from 22 – 34 weeks gestation, compared to placebo:

• Improves obstetric outcome by reducing the incidence of preterm delivery (before 34 weeks gestation).

• Improves neonatal outcome by reducing a composite of death and major morbidity.

• Leads to improved childhood cognitive and neurosensory outcomes at two years.

### Centre(s)

More than 60 hospitals, principally in the UK.

### Design

Double blind randomized placebo controlled trial. The study is in two phases, a screening phase and a treatment phase.

### Inclusion and exclusion criteria

#### Screening phase inclusion criteria

High risk for preterm birth (as indicated by AT LEAST ONE of the criteria i-iv) and ALL of the criteria v-vii:

i. History of previous PTB/second trimester loss (≥16 weeks or ≤37 weeks gestation)

ii. Previous preterm premature rupture of the fetal membranes (≤ 37 weeks gestation)

iii. Short cervical length (≤ 25 mm) on ultrasound at 18–0 -24 + 0 weeks gestation

iv. Any cervical procedure to treat abnormal smears i.e. large loop excision, laser conisation, cold knife conisation or radical diathermy

v. Gestation established by scan at ≤ 16 weeks to ensure that the estimated date of delivery is accurate or the consultant must be confident that the gestation dates are accurate.

vi. Signed Consent form

vii. 16 years of age or older.

#### Treatment phase inclusion criteria

• All women fulfilling the inclusion criteria for the screening phase and who also have a positive screening (fFN) test at 22 + 0 weeks, will be eligible for the main (treatment) phase of the study – these women are subsequently referred to as the “fFN positive” group.

• Additionally, in September 2010, those who have a previous spontaneous labour resulting in a preterm birth ≤ 34 weeks gestation (delivery by any mode) or a short cervix in index pregnancy, defined as cervical length ≤ 25 mm at 18–0 -24 + 0 weeks gestation also became eligible for randomisation even with a negative fFN test. These women are subsequently referred to as the “fFN negative” group

### Exclusion criteria

• Known significant congenital structural or chromosomal fetal anomaly

• Known sensitivity or listed contraindication to progesterone (known allergy or hypersensitivity to progesterone, severe hepatic dysfunction, undiagnosed vaginal bleeding, mammary or genital tract carcinoma, thrombophlebitis, thromboembolic disorders, cerebral haemorrhage, porphyria) or intolerance to progesterone or excipient (including peanut allergy prior to February 2011 given that peanut oil was the excipient in doses issued to participants until November 2010)

• Suspected or proven rupture of the fetal membranes at the time of recruitment

• Multiple pregnancy

Prescription or ingestion of medications known to interact with progesterone (e.g. Bromocriptine, Rifamycin, Ketoconazole or Ciclosporin)

• Women currently prescribed progesterone or who have taken progesterone beyond 18 weeks gestation.

*Intervention(s)* Progesterone (Utrogestan) 200 mg soft capsules or placebo will to be inserted once daily vaginally at bedtime from 22^+0^ - 24^+0^ up to 34^+0^ weeks gestation.

### Randomisation

Randomisation will be carried out online via the web portal or via telephone to the central randomisation facility based at the Robertson Centre for Biostatistics, at the Glasgow Clinical Trials Unit, University of Glasgow.

### Concealment of allocation

Concealment of allocation will be achieved by randomising participants to active or placebo capsules. Placebo capsules will appear identical to active treatment. The outcomes will be measured blind to the allocation.

### Primary and any secondary endpoint(s)

Primary endpoints:

• Obstetric: delivery <34 completed weeks of gestation (Yes/No), (<34 + 0 weeks: outcome of the treatment phase)

• Neonatal: a composite of death or two markers of neonatal morbidity – bronchopulmonary dysplasia in children born at <32 weeks of gestation and brain injury on cerebral ultrasound.

• Childhood: The Bayley III cognitive scale standardised score at two years of chronological age (with an aim to test between 22 months to 26 months - as this is age-standardised all assessments will be valid)

Secondary endpoints:

• Gestation at delivery

• Fetal or neonatal/infant death after trial entry up to 2 years of age [18].

• Incidence of the individual components of the primary neonatal outcome

• Incidence of other major neonatal complications:

• Level of care days, which includes: days of respiratory support, (Either mechanical ventilation or CPAP) and days of oxygen therapy.

• Surfactant administration

• Necrotising enterocolitis, (medical or surgical treatment of confirmed cases)

• Number of discrete episodes of bloodstream or CNS infection (positive blood [19] or CSF culture)

• Daily level of care [20].

• Composite outcome of death or moderate/severe neurodevelopmental impairment at two years of age, defined as per national recommendations [21].

• Individual components of the disability definition and non-neurological disability as defined [21].

• Strengths and difficulties questionnaire (http://www.sdqinfo.com/)

• Score on the PARCA-r (parent assessment of child abilities revised).

• Women’s perceptions of their treatment.

• Maternal and child adverse events (e.g. operative delivery).

### Side-effects reporting and quantification (e.g, WHO scale)

Participants are instructed to contact their Investigator at any time after consenting to join the trial if any symptoms or side effects develop. In the case of any events the Investigator should initiate the appropriate treatment according to their medical judgment. All adverse events (AEs) and Serious Adverse Events (SAE) that occur after being randomized to treatment must be recorded in detail in the patient notes. SAEs occurring in mother or baby from the time a participant is randomised until 30 days after stopping taking study treatment/placebo or until 28 days after delivery (whichever is the later) should be reported to the co-sponsors using the trial documentation. The standard definition of a serious adverse event will be used [22]. This is any event that: results in death; is life threatening (i.e. the subject was at risk of death at the time of the event; it does not refer to an event which hypothetically might have caused death if it were more severe); requires hospitalisation or prolongation of existing hospitalisation; results in persistent or significant disability or incapacity; is a congenital anomaly or birth defect. Hospitalisations for treatment planned prior to randomisation and hospitalisation for elective treatment of a pre-existing condition will not be considered as an SAE, nor, for the purposes of this study, will the following events: miscarriage, preterm labour / suspected preterm labour, premature rupture of membranes (PROM) / suspected PROM, preterm delivery, preterm delivery in maternal interest, preterm delivery in fetal interest, hospitalisation for pregnancy induced hypertension, hospitalisation for “maternal discomfort”, hospitalisation for “rest”, hospitalisation for “observation” or “monitoring” for which the women are admitted for a period of less than 12 hours; normal” childhood illnesses (including infectious diseases and minor injuries) and complications arising from prematurity.

### Statistical analysis plan, including sample size and power calculations

At the commencement of the study in 2008, we planned to recruit 750 women in the “fFN positive” group. We estimated that such a sample size would provide 80 – 95% power at the 5% level of significance for the primary obstetric outcome, 80% power for the neonatal outcome and 93% power for the childhood outcome (although further dilution of the latter was anticipated after incorporation of the deaths in a two stage statistical model – see below).

In 2010, the study was expanded to allow randomisation of women in the “fFN negative” group after analysis of preliminary (blinded) data in July 2010, together with the result of an HTA funded systematic review on screening for preterm birth [23] showed that our initial selection strategy erroneously missed women at medium to high risk of preterm birth. This change was endorsed by the Trial Steering committee, the MHRA, the ethics committee, and by the funder. The inclusion of women at a lower risk of the primary outcomes necessitated an increase in the sample size. The minimum new sample size is anticipated to be in the order of 1125 (375 fFN positive and 750 fFN negative women), but the proportions of each will dictate the exact sample size, as it is anticipated that at least 2 fFN negative women are required to achieve the event rate predicted in 1 fFN positive woman. Workings for the sample size calculation for the fFN positive group alone, and for the revised combined group of fFN positive and fFN negative women are shown in the Appendix.

### Type of analysis (e.g. intention to treat) and statistical tests

All primary efficacy analyses are carried out on the intention to treat population (ITT). Safety analyses are carried out on the safety population (those who initiated on what study medication they received). Primary analyses are repeated in an exploratory manner on the per protocol population (including [i] compliance within an acceptable range and [ii] fulfilment of inclusion and exclusion criteria).

#### Obstetric outcome

The primary obstetric outcome is delivery or fetal death before 34 completed weeks of gestation based on ultrasound (based on the projected date of delivery estimated from scan in the first trimester). The following null hypothesis will be tested: ‘There is no difference in the incidence of delivery or fetal death before 34 completed weeks of gestation between the group treated with 200 mg / day progesterone and the group treated with placebo from week 22–24 to week 34 of gestation or earlier delivery’.

The outcome is compared between the treatment groups using a logistic regression model including treatment and previous pregnancy of at least 14 weeks. The hypothesis will be tested with a likelihood ratio test.

#### Neonatal outcome

The primary neonatal outcome is a binary outcome indicating whether one of the following has occurred:

• Death

• Brain injury (defined as any intraventricular haemorrhage (IVH) (excludes subependymal haemorrhages), parenchymal cystic or haemorrhagic lesion or persistent ventriculomegaly (VI >97th percentile)

• Severe chronic lung disease (defined as need for ≥30% oxygen and/or positive pressure (positive pressure ventilation or nasal continuous positive airway pressure) at 36 weeks post menstrual age or discharge, which ever comes first).

The following null hypothesis will be tested: ‘There is no difference in the combined incidence of neonatal death, brain injury or severe chronic lung disease between the group treated with 200 mg / day progesterone and the group treated with placebo from week 22–24 to week 34 or earlier delivery’ This outcome is also compared between the treatment groups using a logistic regression model including treatment and previous pregnancy of at least 14 weeks. The hypothesis will be tested with a likelihood ratio test.

#### Childhood outcome

The primary childhood outcome is the Bayley III score, a continuous measure. This outcome will, by definition, not be available on babies who have died. Thus deaths need to be incorporated into the analysis, since the number of deaths may be sufficiently large as not to be negligible, and/or there may be a difference in the number of deaths between the two randomised groups. We will therefore use a two-stage statistical model that jointly models the treatment effect in both deaths and survivors [24], with deaths modelled using a binomial test and survivors modelled using a generalised linear model. The two parts are then combined to form the appropriate test statistic. Secondary analyses that adjust the estimated treatment effect for covariates felt to be of importance will be used as appropriate. Note that we will not be adjusting for gestational age in our analysis of childhood outcomes. The hypothesised mechanism of action of progesterone is to increase gestational age by reducing the proportion of women giving birth prematurely. To adjust for a post randomisation covariate (gestational age) which is a direct measure of the treatment effect, in a model that is estimating the consequence of that treatment effect (in terms of developmental outcome) is not statistically sound.

A fully detailed Statistical Analysis Plan will be prepared prior to unblinding.

### Planned subgroup analyses

In order to determine whether a reduced or improved response to progesterone can be predicted, subgroups of the ITT population will be formed according to the following factors:

• reason for risk of preterm delivery (spontaneous preterm birth yes / no and any preterm birth yes / no)

• previous pregnancy of at least 14 weeks (yes / no)

• cervical length at 18–24 weeks gestation (≤25 mm / >25 mm and ≤15 mm / >15 mm)

• chorioamnionitis diagnosed on pathology (yes / no)

### Ethical issues, including: Ethics committee approval

The study has been approved by the Scotland (A) Research Ethics Committee, reference number 08/MRE00/6.

### Interim analyses and stopping rules

Interim unblinded analyses will be performed on safety, efficacy and possibly futility criteria for the purposes of review by the independent Data Monitoring Committee (iDMC) only. Masking will be maintained and analysis of obstetric and neonatal outcomes will be deferred until conclusion of the two-year childhood assessments. Thus no interim analyses will be revealed to investigators, participants, or anyone other than the DMC members (and statistical advisors) until completion of the two-year childhood assessments.

### Committee oversights

There is an independent Trial Steering Committee and independent Data Monitoring Committee.

### Indemnities

The sponsor has clinical trial insurance which covers this study. This insurance includes indemnification for the TSC and DMC. There is no “no fault” indemnity.

### Publication plan

The results of the trial will be published in peer reviewed journals. The results of the obstetric and neonatal outcomes will not be published until data collection for the childhood follow up part of the study is complete.

### Funding

The study is funded by the MRC/EME -funder reference G0700452, grant No: 84982

### Start date

Recruitment began in January 2009, as of May 2012, over 800 women had been randomized

### End of data collection

Estimated October 2015

### Reporting date

Estimated mid 2016

### Trial registration

ISRCTN14568373

## Discussion

Despite the many publications on the use of progesterone for the prevention of preterm birth, we believe that that further data, particularly on the long term effect for the baby, is needed before the use of progesterone can be routinely recommended in pregnant women at high risk of preterm birth. Many authorities, including the FDA^a^ and the Royal College of Obstetricians in the UK^c^ agree. The Society of Obstetricians and Gynecologists of Canada (SOGC) [25] suggests that women at risk of should be encouraged to enroll in trials of progesterone, although the SOGC also gives advice about the dose and formulation of progestogens for women who have opted to take them. Other authorities endorse the use of progesterone based on current literature. For example the American College of Obstetricians and Gynecologists, in guidelines published in 2008 and reaffirmed in 2011 [26] is more positive about the use of progesterone, saying that “progesterone supplementation for the prevention of recurrent preterm birth should be offered to women with a singleton pregnancy and a prior spontaneous preterm [before 37 weeks] birth” and that “progesterone supplementation for asymptomatic women with a very short cervical length (less than 15 mm) may be considered”. In 2012, the Society of Maternal and Fetal Medicine Publications committee suggests that women with singleton pregnancies with prior preterm birth be given 17 alpha hydroxyprogesterone, and that those with a short cervix ≤ 20 mm be given vaginal progesterone (although they indicate that routine screening is “an object of debate”) [13].

The OPPTIMUM study will provide further evidence on the effectiveness of vaginal progesterone for the prevention of preterm delivery in selected high risk women. Crucially, it will also determine whether prevention of preterm birth by progesterone prophylaxis is associated with long term benefit for the baby.

## Appendix

### Sample size calculations

#### Original sample size calculation based on data in the literature on the “fFN +” group

A sample size of 750 (375 per group) gives adequate statistical power to detect clinically important and plausible differences in the three primary measures of outcome. All these power calculations allow for loss to follow up rates and compliance. Delivery <34 weeks on placebo is expected to be 40% (data from an untreated high risk UK population with a positive fFN test at 22 weeks [27] and 27% on progesterone consistent with the odds ratio of 0.45 for the overall PTB with any progestational agent in the most recent systematic review [28]. With 750 randomised, the study will have 95% power at a 5% level of significance to detect such a reduction from 40% to 27% using a two-sided binomial test. For a more modest reduction from 40% to 30% (odds ratio 0.64) the study would still have 80% power. For the neonatal outcome, the Meis study found 7.2% died, 14.6% had CLD and 5.2% had IVH on placebo, compared to 4.6% death, 8.6% CLD and 1.3% IVH on progesterone [4]. Our neonatal primary outcome is a composite of these and also includes non-haemorrhagic brain injuries. With n = 750 randomised, the OPPTIMUM study would have 80% power at a 5% level of significance to detect a difference in this composite outcome of death, brain damage, or chronic lung disease from 20 to 12%, using a binomial test.

The childhood developmental outcome at 2 years will be assessed using the Bayley III Cognitive Scale which correlates well with later IQ. With 750 randomised, the study will have 93% power at a 5% level of significance to detect a difference in means equivalent to 0.25 of a standard deviations, using a two sample two sided *t*-test. Based on previous work [29], we estimate the standard deviation will be about 15 points, enabling us to detect a difference of 4 points in the Bayley Score. In clinical terms, a difference of 4 points is small, thus the power of the study to detect larger, more clinically significant differences, is high. We have powered this outcome at over 90% to make some allowance for a possible dilution of the power due to incorporating the deaths (using a two stage statistical model).

#### The revised sample size calculation below, based on blinded data (as of April 2011) and inclusion of the "fFN"- group is around 1250 participants

**Obstetric outcome** (delivery <34 weeks).

The table below gives the estimated power for three combinations of sample size (all 1:2 fFN+/fFN- groups, assuming 375:750 (total 1125), 400:800 (total 1200) and 425:850 (total 1275) assuming (fFN+, fFN-) event rates (delivery < 34 weeks) of (40%, 10%), (45%, 13%), and (45%, 15%). All calculations assume a relative treatment effect of 32.5%.

The justification for these combinations is as follows:

1. The 40% untreated rate is as per the original assumption. We have looked at the blinded (aggregated) event rate for the first 67 women randomised in OPPTIMUM (ignoring any possible bias created by recording the early births before later births in the most recently randomised). For the fFN + group, 29/67 (43%, 95% exact confidence interval 31% to 56%) had delivered at <34 weeks. The blinded event rate according to the assumed 40% untreated rate with a 27% treated rate (32.5% relative reduction) would be 33.5%. Although the numbers are relatively small, there appears to be a higher event rate than anticipated, which would be consistent with having selected a higher risk and hence scarcer subset than we planned for). An overall blinded rate of 43% could comprise an untreated rate of 52% and a treated rate of 35%. We assume conservatively that the baseline event rate in the untreated fFN + group is 45%.

2. For the fFN- group women data on the first 24 women suggested that 8/24 (33%, exact 95% confidence interval 16% to 55%) had delivered early. As indicated above, this percentage is likely to be inflated since we record the early deliveries before observing the term deliveries. However, fortunately, we also had followed up those who were not randomised (by definition, all of whom had a negative fFN test) into OPPTIMUM from the start. Of the 398 followed, 32 have had a delivery <34 weeks. We know that 57% of these 398 would have been eligible for the trial (short cervix < 25 mm), and the epidemiology indicates that it is very unlikely to give birth <34 weeks with none of the classic risk factors. So we conservatively estimate that around 13% (i.e. around 29/225, exact confidence interval 9% to 18%) of those recruited into the fFN- group, based on OPPTIMUM data, will have a delivery <34 weeks.

The conclusion is that it seems likely that if we recruit at least 1125 (375:750) we will have almost 90% for the primary obstetric outcome, assuming a slightly higher rate in the fFN + group (45% rather than 40%) and a corresponding untreated event rate of 13% in the fFN- women. If as we expect from the early returns the fFN- event rate is >15%, we will be comfortably powered at the original 95% for the obstetric outcome. Table 1 shows the various power scenarios.

**Table 1:** Power scenarios for the neonatal outcome

**fFN+**	**fFN-**	**Subjects (fFN+/fFN-) [Total]**	**Power**
40%	10%	375/750 (1125)	81%
		400/800 (1200)	83%
		425/850 (1275)	85%
45%	13%	375/750 (1125)	88%
		400/800 (1200)	90%
		425/850 (1275)	92%
50%	15%	375/750 (1125)	93%
		400/800 (1200)	94%
		425/850 (1275)	95%

**The neonatal outcome** (neonatal death, severe chronic lung disease, intraventricular haemorrhage)**.**

Data from the first cohort of women randomised (and in particular those randomised in the low risk group) into OPPTIMUM was even sparser for this outcome, as it is determined by convention to up to 30 days after a full term birth. For all those randomised with mature data at the time of analysis, 11/68 have had the composite neonatal outcome (16%, exact 95% confidence interval of 8% to 27%). Given that the rate of pre-term birth is higher than anticipated, it seems reasonable to inflate the assumed neonatal event rate from 20% to 25% (giving an blinded event rate of 20%, assuming a 40% relative reduction – and 20% is very consistent with the observed 95% confidence interval of 8% to 27%). If we then further assume a pro-rata event rate in line with the rates of delivery <34 weeks on the obstetric outcome, with the fFN- having roughly one-third the events of the fFN + i.e. 8% rather than 25%, then Table 2 below shows the various power scenarios:

**Table 2:** Power scenarios for the obstetric outcome

**fFN+**	**fFN-**	**Subjects (fFN+/ fFN-) [Total]**	**Power**
25%	8%	375/750 (1125)	81%
		400/800 (1200)	83%
		425/850 (1275)	86%

We conclude that by randomising at least 375 fFN + and 750 fFN- women, assuming an untreated rate of 25% of the composite of neonatal death, severe chronic lung disease, intraventricular haemorrhage in the fFN+, and 8% in the fFN-, with an assumed 40% relative treatment effect (as per original calculation) the original power of the study for this neonatal outcome will be preserved at >80% power.

**The childhood development outcome** (Bayley Score at 2 years).

There are no data yet on this outcome within OPPTIMUM. In the original calculation we assumed that the difference in mean Bayley score would be 4 units with a common standard deviation of 15 units. It is more difficult to assess the power convincingly with a mixture of fFN + and fFN- women on a continuous outcome such as the Bayley Score, since the power calculation requires assumptions about not just the anticipated treatment effect but also the assumed variability via the standard deviation. If we assume the same 4 unit difference in the fFN + and a 4/3 unit difference in the fFN- group (consistent with the pro-rata rate of delivery <34 weeks), with the same 15 unit standard deviation, then the study will have 71%, 73%, and 76% power if 1125, 1200, or 1275 are randomised (375:750, 400:800; and 425:850) as per Tables 1 and 2 above. However, this is for an unadjusted analysis, and in practice we will adjust for fFN + and fFN- group, and a limited number of other baseline covariates strongly related to Bayley Score at 2 years (e.g. gender) as specified in the statistical analysis plan, and this will reduce the variability and hence increase the power. For example, if the underlying variability in the lower risk group is lower – say halved, at 7.5 units, consistent with a higher proportion having uniformly high Bayley Scores since they have no disability – then the approximate power would be 93%, 94%, and 95% respectively for 1125, 1200, or 1275 randomised in total. In practice the reduction in variability by adjusting for both this design variate (fFN + and fFN- ) and additional baseline covariates may be considerably greater, so we are confident that the original power on the childhood development outcome will be protected at or above the original 90% level by randomising at least 1125 subjects (375:750).

## Endnotes

^a^http://www.fda.gov/NewsEvents/Newsroom/PressAnnouncements/ucm242234.htm

^b^http://www.cbsnews.com/8301-505245_162-57363066/fda-panel-votes-down-preterm-birth-gel/

^c^http://www.rcog.org.uk/womens-health/guidelines/use-progesterone-prevent-preterm-delivery

## Competing interests

JEN has received research grants from (non commercial) funding agencies for pregnancy related conditions – full list available on request, was a paid consultant to a small drug company (Preglem) with an interest in obstetric /gynaecological drugs - < £5,000 2010 – 2012, and an unpaid consultant to Hologic (who manufacture fibronectin amongst others).

ST has received research grants from commercial and non-commercial funding agencies for pregnancy-related research. Full list available on request. ST is paid or unpaid consultant for GSK, Ferring and Hologic.

PB has acted as a consultant for GSK.

AS receives funds for educational purposes from Hologic, Ferring, GSK and Alere. He sits on advisory boards for Roche, GSK and Hologic. AS receieves research funds from Alere and Hologic which are paid to his institution.

## Author’ contributions

The protocol was jointly devised by all the authors with the exception of SW. JEN prepared the first draft of this manuscript for submission. All authors commented and agreed the final draft.

## Disclaimer

This report is independent research funded by the MRC and managed by the NIHR on behalf of the MRC-NIHR partnership. The views expressed in this publication are those of the author(s) and not necessarily those of the MRC, NHS, NIHR or the Department of Health.

## Pre-publication history

The pre-publication history for this paper can be accessed here:

http://www.biomedcentral.com/1471-2393/12/79/prepub
